# Corrigendum: (1) Some OFF Bipolar Cell Types Make Contact With Both Rods and Cones in Macaque and Mouse Retinas; (2) OFF Bipolar Cells in Macaque Retina: Type-specific Connectivity in the Outer and Inner Synaptic Layers

**DOI:** 10.3389/fnana.2015.00144

**Published:** 2015-11-13

**Authors:** Yoshihiko Tsukamoto, Naoko Omi

**Affiliations:** ^1^Studio RetinaNishinomiya, Japan; ^2^Department of Biology, Hyogo College of MedicineNishinomiya, Japan

**Keywords:** monkey retina, basal synapse, ribbon synapse, neural circuits, serial section electron microscopy

In the upper panel of **Figure 2** of article (1), and in the lower panel of Figure 1 of article (2), the side-view picture in the position of type DB3b cell 3 (DB3b-3) is wrong. This picture was mistakenly replaced with type DB2 cell 3 (DB2-3). Revised figures appear below. Also in the legend of Figure 2, INL should be “inner nuclear layer,” instead of “inner plexiform layer.” The top-view pictures of the axon terminal and dendrites of cell DB3b-3 in other figures and their morphological measurements in the text are all correct. Results and conclusions are unaffected.

**Article (1)**

**Figure 2:**
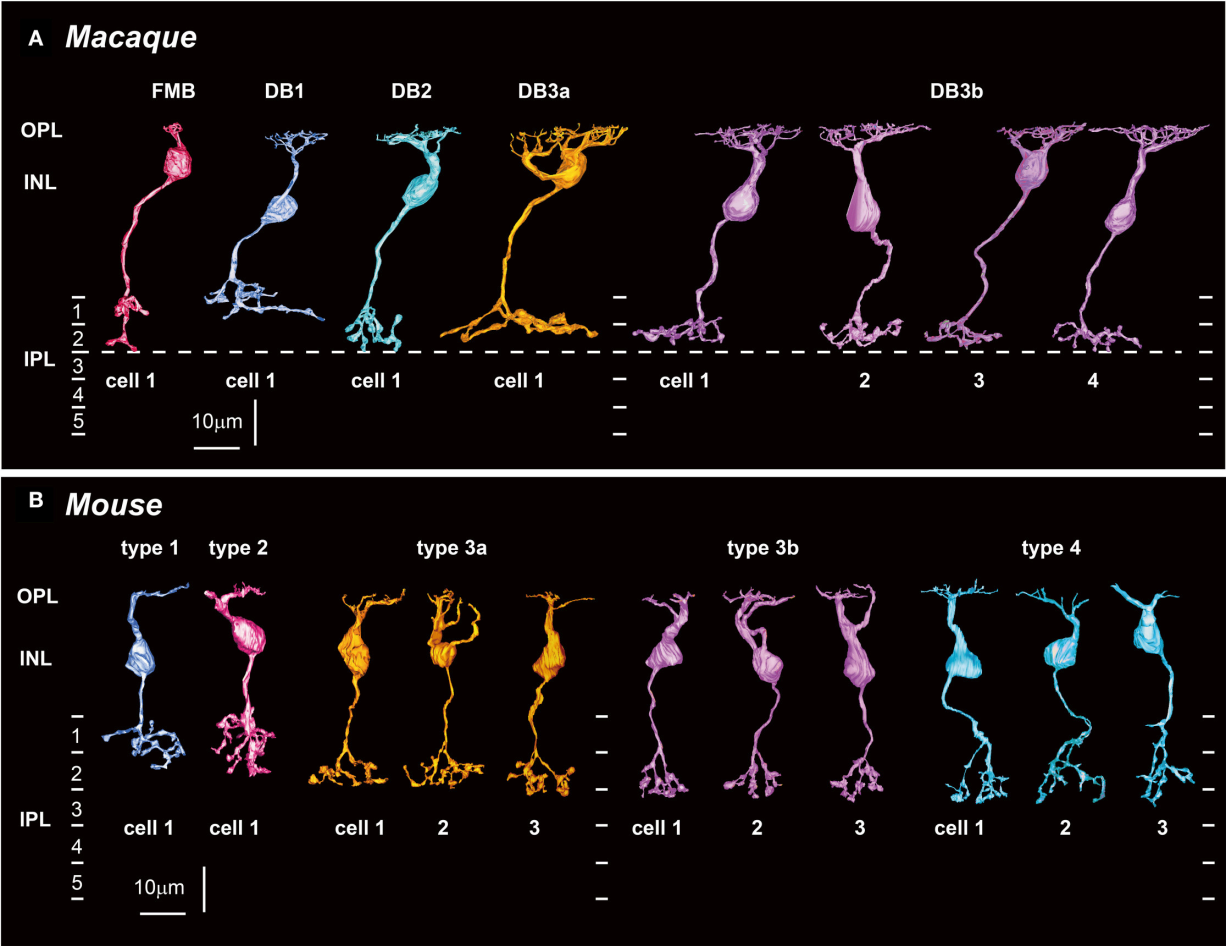
**Five types of OFF bipolar cells in macaque (A) and mouse (B) retinas. (A)** FMB, DB1, DB2, and DB3a cells (one example of each; left) and DB3b cells (four examples, DB3b-1 to DB3b-4) that make contact with both rods and cones in the macaque retina (right). **(B)** T1 and T2 cells (one example of each type; left), and T3a, T3b, and T4 cells (three examples of each) that make contact with both rods and cones in the mouse retina (right). Potential homologous pairs between macaque and mouse are depicted using the same colors (such as FMB and T2 in red and DB2 and T4 in light blue). OPL, outer plexiform layer; INL, inner nuclear layer; IPL, inner plexiform layer. Dotted lines indicate 0, 20, 40, and 60% depths in IPL.

**Article (2)**

**Figure 1:**
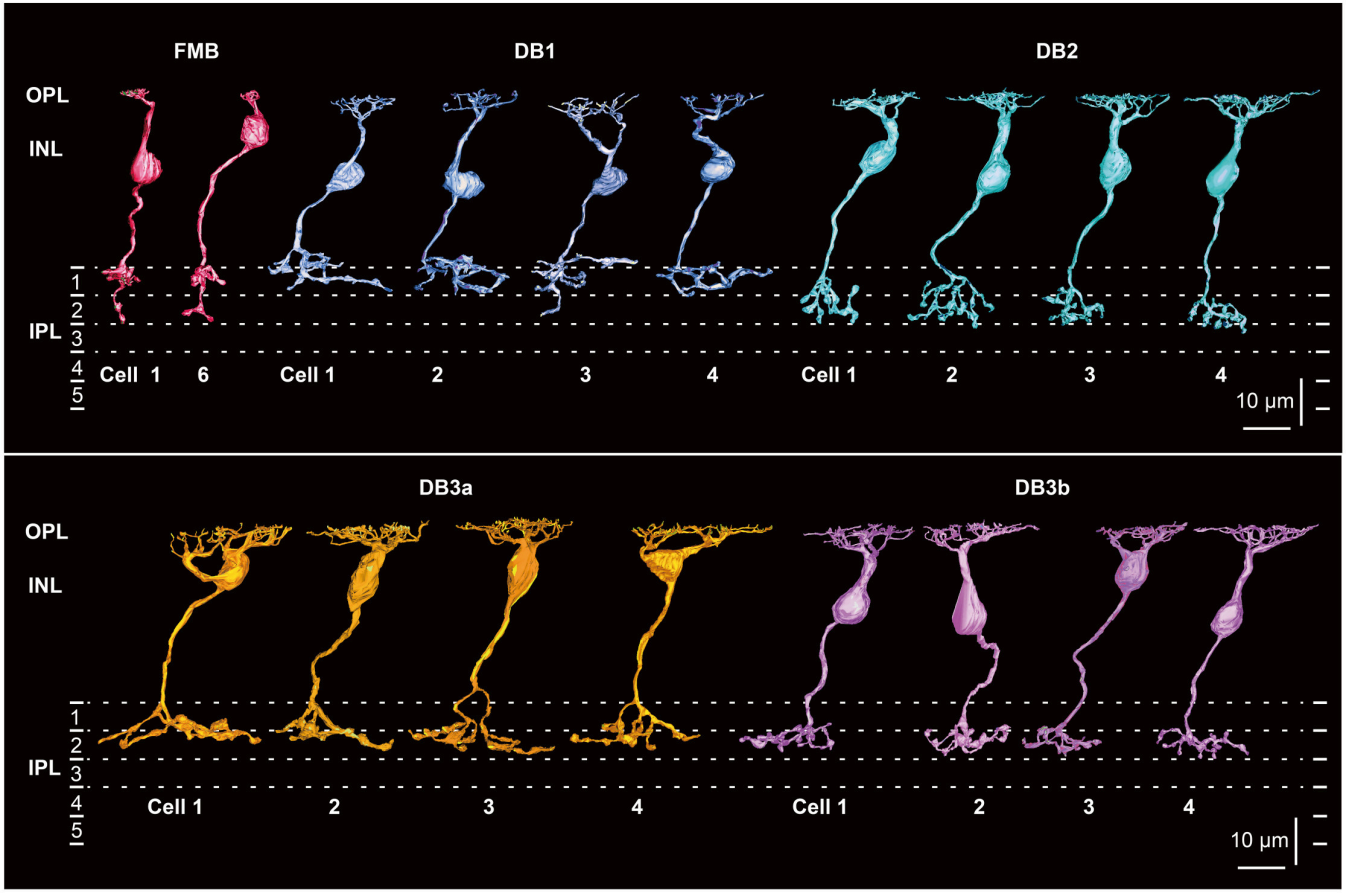
**Morphology and stratification of FMB, DB1, DB2, DB3a, and DB3b types of OFF bipolar cells**. FMB cell-1 and cell-6 (FMB-1 and FMB-6) are connected to M/L and S cones, respectively. Four cells (1–4) are displayed for each DB type. Each stratum of the IPL (1–5) is 6 μm thick. Strata 1–2 comprise the OFF sublamina.

## Author contributions

The authors had full access to all the content in this commentary and take full responsibility for the accuracy of the data. YT wrote the manuscript. NO checked the manuscript.

### Conflict of interest statement

The authors declare that the research was conducted in the absence of any commercial or financial relationships that could be construed as a potential conflict of interest.

